# The relationship between the decrease in haemoglobin concentration and the volume of fluids administered during resuscitation from septic shock may not be so “weak”

**DOI:** 10.1186/s13054-018-2118-6

**Published:** 2018-09-20

**Authors:** Azriel Perel

**Affiliations:** Anesthesiology and Intensive Care, Sheba Medical Center, Tel Aviv University, Tel Aviv, Israel

In a retrospective analysis of the database of the ARISE trial [1], Maiden et al. [2] found an independent though weak association between haemoglobin concentration (Hgb) and the volume of fluids administered during resuscitation from septic shock. The volume infused accounted for less than 20% of the observed decrease in the Hgb, but the explanations for this decrease, other than haemodilution, seem unlikely even to the authors themselves [2]. A closer look at this study suggests a much stronger association between the volume infused and Hgb decrease.

Of the 1600 ARISE patients, Maiden et al. excluded 281 who received blood transfusions [2]. It may well be that the transfused patients were those with the greatest degree of haemodilution, the low Hgb prompting the decision to transfuse. The data for these excluded patients might have shown a stronger association between volume infused and Hgb decrease. Of note, significantly more patients were transfused during the first 6 h in the EGDT group of the ARISE trial compared with the usual-care group [1]. A significant association between the amount of fluids and early blood transfusions was also recently reported in septic patients without shock [3].

**Fig. 1:**
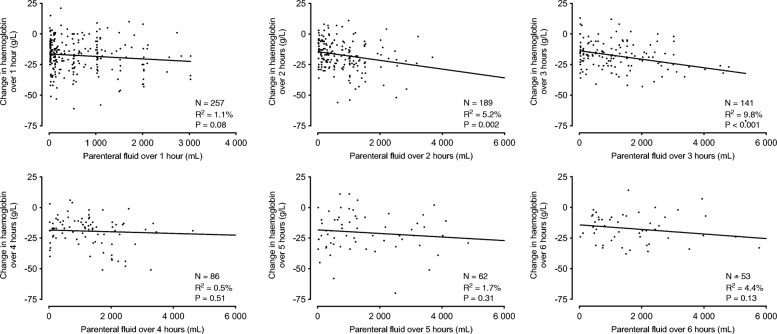
Figure S3 of [2], reproduced with permission

Although the ARISE trial was not aimed at reporting Hgb values during the first 6 h of resuscitation, Maiden et al. report such values from roughly 200 patients [2]. Many of these patients seem to have had no fluids administered during the first hours, and yet have a significant decrease in Hgb of more than 2 g/dL (Fig. 1). However, all patients in the ARISE trial received about 35 ml/kg prior to randomisation [1], a fact that explains the initial observed decrease in Hgb in Maiden et al.’s study [2]. Taking this significant amount of fluids into consideration when calculating the association between the infused fluids and Hgb decrease would have made this association much stronger.

Iatrogenic haemodilution is a possible complication of fluid administration [4]. Maiden et al. do indeed show that a greater decrease in Hgb was independently associated with increased duration of ventilation, length of ICU and hospital stay, and mortality [2]. When large amounts of fluids are given in order to maximise oxygen delivery, the resulting haemodilution may also lead to avoidable blood transfusions [5]. The data presented by Maiden et al. [2] do show that this is a real possibility during early resuscitation from septic shock.
