# Supplementary material for: The relationship between the decrease in haemoglobin concentration and the volume of fluids administered during resuscitation from septic shock may not be so “weak”
Source: Crit Care. 2018 Sep 20;22:234. doi: 10.1186/s13054-018-2118-6 (PMC6148991; doi:10.1186/s13054-018-2118-6)
Supplement: Related texts

# Reply: Authors’ response

**Authors:** Matthew J. Maiden, Mark E. Finnis, Sandra Peake, Simon McRae, Anthony Delaney, Michael Bailey, Rinaldo Bellomo

Intravenous fluids given to healthy volunteers [6] or administered during surgical procedures have been shown to cause haemodilution [5]. Hence, we too were somewhat surprised to observe a relatively weak (but statistically significant) association between the change in haemoglobin (Hb) concentration and volume of intravenous fluids administered to patients resuscitated from septic shock. However, intravenous fluid may not induce the same degree of haemodilution in septic patients as healthy volunteers or elective surgical patients, particularly given the intra- and extra-vascular fluid shifts that occur with endothelial dysfunction in sepsis.

We believe it unlikely that we missed identifying a stronger association between intravenous fluid volume and decline in haemoglobin. We analysed patients with complete data for haemoglobin and intravenous fluid volume at each time point in the ARISE trial (*N* = 1011 at 24 h, *N* = 990 at 72 h; Fig. 2 in [2]) and performed a sensitivity analysis using data available during the first 6 h of resuscitation (*N* = 788; Fig. 1) [2]. All patients received intravenous fluids, and haemoglobin declined even in those who received a small volume of fluid. Many clinical factors that may have been confounders for this association were considered, but these had only a marginal influence. Furthermore, our findings are consistent with earlier (albeit smaller) clinical studies investigating the same relationship in critical illness [7, 8].

We are unable to contribute to the hypothesis that patients in ARISE may have been transfused red cells due to intravenous fluid induced haemodilution. Firstly, we considered haemoglobin concentration an independent variable and transfused patients (281/1600, 17.6%) were excluded, as supplementing red cells would have confounded the analysis. Secondly, patients who received red cells were anaemic at study enrolment (median Hb g/L; 98 [83–116] vs 133 [118–146], *P* < 0.001) while having received similar volume of fluids prior to enrolment (median L; 2.5 [1.7–3.3] vs 2.4 [2.0–3.3], *P* = 0.29). Thirdly, when analysing the entire ARISE cohort and incorporating transfusion in multivariate analysis, the decline of haemoglobin with fluid volume was only marginally greater than described in the original analysis (change in Hb over 24 h, − 1.6 g Hb per litre of fluid, 95% CI − 1.2 to − 2.0, R^2^ = 11%, *P* < 0.001; change in Hb over 72 h, − 1.6 g Hb per litre of fluid, 95% CI − 1.3 to − 1.9, R^2^ = 26%, *P* < 0.001; Table 3 in [2]) [2]. Finally, incorporating the fluid volume administered prior to randomisation into this analysis made the association between haemoglobin and fluid volume weaker (change in Hb over 24 h, − 1.2 g Hb per litre of fluid, 95% CI − 0.9 to − 1.6, *P* < 0.001). The evidence that intravenous fluid causes clinically meaningful haemodilutional anaemia in septic shock remains weak, and other causes of the decline in haemoglobin should be considered.
